# HIV Patient With a Complicated and Unique Presentation of the Monkeypox Virus

**DOI:** 10.7759/cureus.44607

**Published:** 2023-09-03

**Authors:** Prachi Patel, Bhavi Purohit

**Affiliations:** 1 Internal Medicine, Philadelphia College of Osteopathic Medicine, Atlanta, USA; 2 Internal Medicine, Wellstar North Fulton Hospital, Roswell, USA

**Keywords:** monkeypox virus, painful skin lesions, pulmonary complications, people living with hiv/aids, immunocompromised patient

## Abstract

Monkeypox virus presents a concerning challenge, as it is known for its ability to spread through a diverse range of transmission routes, including established pathways like direct contact and shared living spaces, with the spectrum of potential methods appearing to be limitless. This immunocompromised 43-year-old HIV patient presented to the emergency department (ED) in the United States with shortness of breath, cough, and painful lesions for several months. Numerous consults and a full panel of testing revealed an odd case of the monkeypox virus that strayed away from the predefined classifications for the disease. The case sheds light on the need for further research in immunocompromised patient populations as their presentations may be different from what research currently suggests.

## Introduction

Monkeypox is a zoonotic orthopox virus from the Poxviridaefamily hosts within animal reservoirs such as pigs, mice, monkeys, and rats. This virus is thought to have originated in Africa and spread from animals to humans and then from humans to humans via a variety of transmission routes: feces, respiratory droplets, bodily fluids, and direct contact [1]. The disease has become more common in younger individuals under the age of 40 and predominantly in men who have sex with other men [2].

Monkeypox initially presents with fever, fatigue, and lymphadenopathy and then slowly progresses to mucosal lesions and concentrated, circumscribed, and umbilicated skin lesions. There is epidermal central necrosis that extends into the superficial layers of the dermis causing all phases of the rash to be painful. The rash usually first appears on the face and then distributes centrifugally to other parts of the body such as the extremities rather than centrally at the abdomen and trunk [3]. The natural progression of the monkeypox lesions goes enanthem, macular, papular, vesicular, pustular, crusted, and desquamation [2].

Complications of monkeypox include skin scarring, corneal scarring, pneumonia, sepsis, and others and treatment options include supportive management [1]. After reaching the cervical and inguinal lymph nodes, the virus can circulate to other organs such as the lungs, gastrointestinal tract, and eyes. Gastrointestinal symptoms include nausea, vomiting, and diarrhea, while corneal scarring can lead to vision loss [2].

## Case presentation

A 43-year-old male patient presented to the emergency department (ED) with shortness of breath and a cough for several weeks. His vitals were a blood pressure of 94/62 mmHg, heart rate of 108 beats per minute, SpO_2_ of 90%, respiratory rate of 20 breaths per minute, and a BMI of 17.94 kg/m^2^. Along with the pulmonary symptoms, he had several painful lesions throughout his body for the past couple of months. The patient had been diagnosed with HIV along with secondary neurosyphilis a few years prior and had a smoking and drug abuse history including methamphetamine use two weeks prior. He was retested and negative for syphilis but had also been noncompliant with retroviral therapy for the past six months.

Physical exam findings showed general fatigue, weakness, and bilateral crackles. Further examination revealed several painful, well-circumscribed, crusted lesions with central erosion. These lesions were located on the right lateral eyebrow, scalp, left lateral shoulder, and lower left back, along with multiple open confluent perianal lesions in the intergluteal cleft with surrounding erythema. No lesions were present on the palms or soles nor were there genital or oral ulcers. 

The patient was confirmed as immunocompromised with his CD4 count of 21 cells/mm^3^ and viral load of 475,000 per milliliter of blood. A dermatology consult first ruled out cytomegalovirus, cryptococcus, aspergillosis, Kaposi sarcoma, syphilis, cutaneous tuberculosis, and Mycobacterium with a negative shave biopsy and Legionella and *Streptococcus pneumoniae* with a negative antigen urine test. The infectious disease department was then consulted, and they recommended urine tests for gonorrhea and chlamydia, a* Pneumocystis jirovecii* pneumoniae smear, a rapid plasma reagin (RPR) test, and anal wound culture for aerobes and anaerobes, all of which resulted in negative. Along with the comprehensive dermatological and infectious workup, thorough bloodwork was completed with unimpressive results. Finally, a conclusive virus DNA PCR was positive for monkeypox.

The hospital team started him on a combination therapy of tecovirimat and cidofovir as a treatment for the monkeypox virus in highly immunocompromised patients. He was also restarted on his antiretroviral medications and given lidocaine and morphine to manage his severe lesional pain. Though the recommendation was to stay in the hospital under isolation precautions for two weeks, the patient left against medical advice. He was followed up by the infectious disease department, his primary care physician, and the local health department outpatient to continue monitoring his symptoms and manage his antiretroviral medications. 

## Discussion

**Figure 1:**
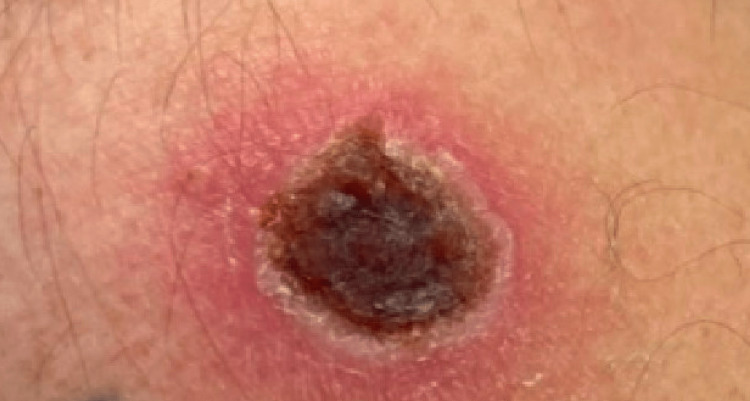
3 cm monkeypox lesion on the left shoulder

**Figure 2:**
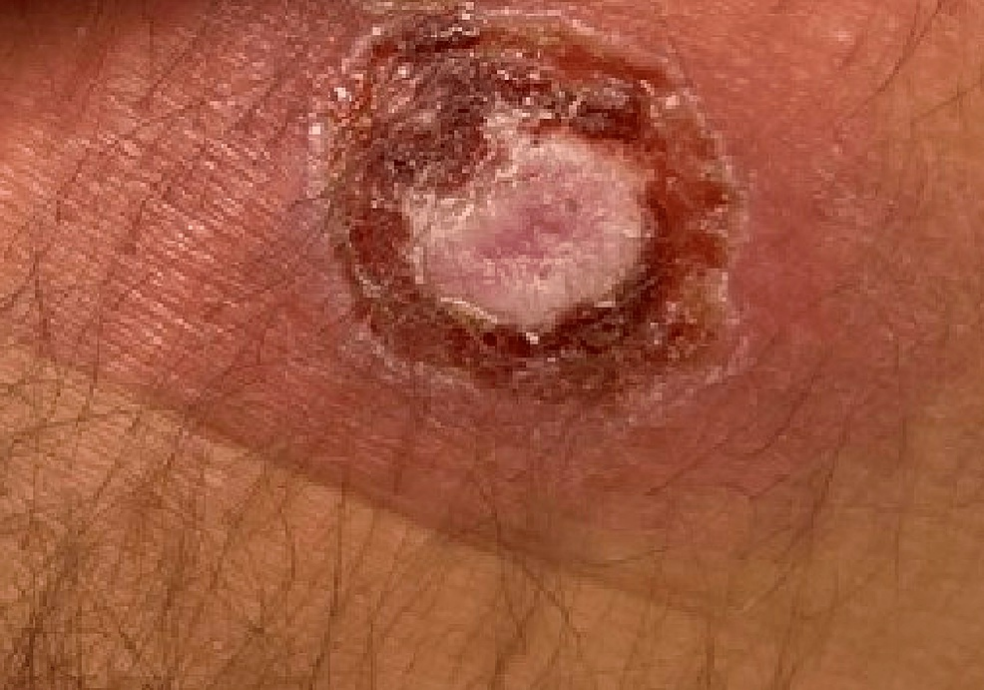
2.5 cm monkeypox lesion on intergluteal cleft

This patient had unusual features associated with this virus; therefore, he was originally initiated on airborne precautions with his ambiguous symptoms before the monkeypox virus was confirmed. After diagnosis, he was placed on contact precautions in a single-person room where healthcare personnel wore gloves, gowns, eye protection, and N95 masks before entering the room. There are standard presentations common to monkeypox that this patient steered away from causing the diagnosis process to be much longer than anticipated. Monkeypox has a classic type of rash characteristic to it which is the centrifugally located umbilicated lesion. This patient’s rash was far from that description when he presented to the ED as well as months prior to that when he first noticed a rash. The rash did not have an umbilicated appearance and was centrally located on the trunk and abdomen rather than on the face or extremities. Monkeypox lesions generally range in size from 2 mm to 10 mm (Figure 1 and Figure 2), but this patient had multiple lesions that went far beyond the range and were up to 2 cm to 5 cm in size [1]. What does correlate with the normal presentation is the excruciating pain that the patient dealt with from the rash.

The virus spreads via lymph nodes, and it is reported that 84% of patients with monkeypox have lymphadenopathy. However, this patient not only did not have lymphadenopathy but also did not have the fever that 36% of the patient population has [4,5]. Research shows that HIV-positive patients also infected with monkeypox demonstrate a prominence of specific symptoms unique to them compared to other non-immunocompromised monkeypox patients. Though there is increased frequency for perioral lesions and other manifestations such as pharyngitis in 17%, proctitis in 12%, and penile/scrotal edema in 12% of the HIV population, this patient did not showcase any such symptoms further deviating from the norm [4].

**Figure 3:**
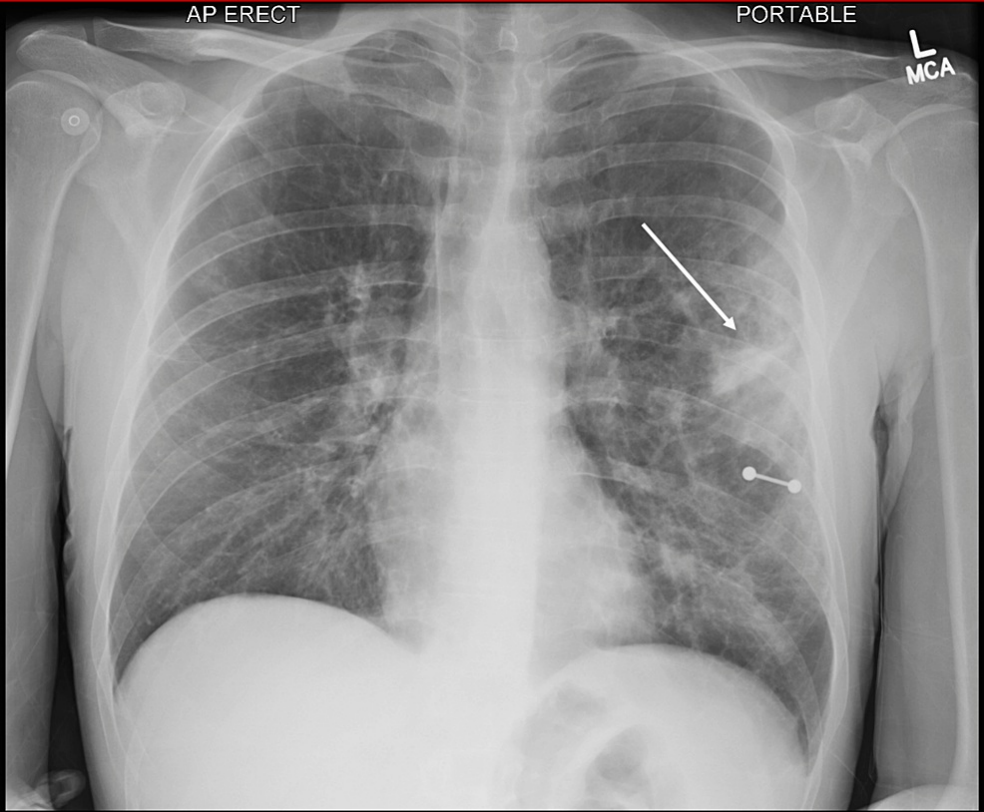
Chest X-ray showing multifocal bilateral parenchymal opacities greatest in the left upper lobe

**Figure 4:**
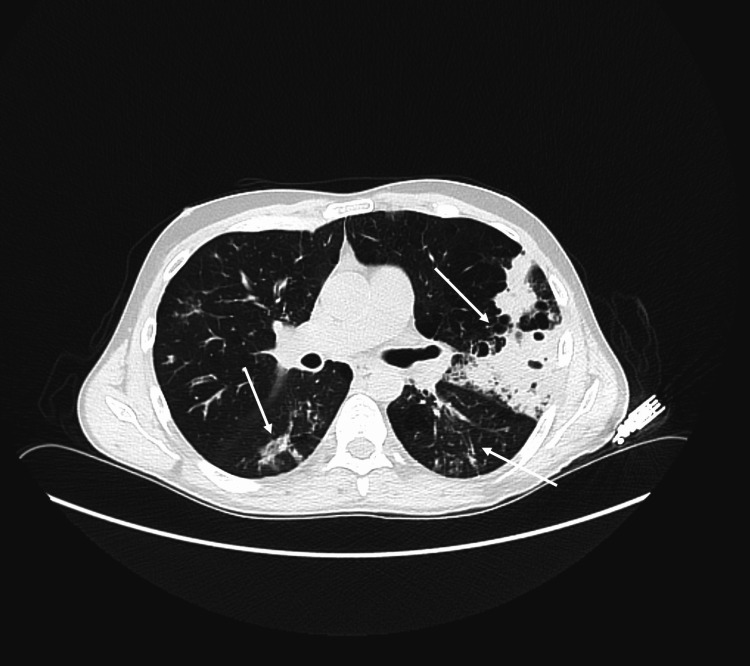
CT chest with contrast portraying focal and patchy consolidations, centrilobular nodules, multifocal pneumonia, and emphysema in both lung lobes

Pulmonary complications in monkeypox are more often seen in patients co-infected with the influenza virus [2]. Though this patient was negative for influenza, he was immunocompromised thus putting him at higher risk for pulmonary distress. His chest x-ray portrayed multifocal parenchymal opacities greatest in the left upper lobe (Figure 3), and CT with contrast provided further details such as the focal consolidation in the posterior aspect of the left upper lobe with additional patchy consolidations and centrilobular nodules within the left lower lobes and right upper lobe consistent with multifocal pneumonia with hyperinflation and emphysema (Figure 4). Studies have shown that people with HIV and monkeypox are 8% more likely to be hospitalized than those without HIV due to high rates of complications and mortality risks [5].

## Conclusions

Monkeypox currently has no treatment besides supportive care on a symptomatic basis for an immunocompetent patient. Though this might be a concrete management plan for a healthy patient, it emphasizes the need for more research in immunocompromised patients who also have monkeypox as they have significantly increased risks for complications and mortality. Their plan of care should be different from the average population with this disease. With the relatively new outbreak of monkeypox in the Western hemisphere, there are bound to be unusual cases similar to this patient brought to attention. Therefore, it grants a prompt and diversified knowledge base to stem patient care from.
